# Genic and Intergenic SSR Database Generation, SNPs Determination and Pathway Annotations, in Date Palm (*Phoenix dactylifera* L.)

**DOI:** 10.1371/journal.pone.0159268

**Published:** 2016-07-19

**Authors:** Morad M. Mokhtar, Sami S. Adawy, Salah El-Din S. El-Assal, Ebtissam H. A. Hussein

**Affiliations:** 1 Molecular Genetics and Genome Mapping Lab., Agricultural Genetic Engineering Research Institute, ARC, Giza, Egypt; 2 Genetics Department, Faculty of Agriculture, Cairo University, Giza, Egypt; USDA/ARS, UNITED STATES

## Abstract

The present investigation was carried out aiming to use the bioinformatics tools in order to identify and characterize, simple sequence repeats within the third Version of the date palm genome and develop a new SSR primers database. In addition single nucleotide polymorphisms (SNPs) that are located within the SSR flanking regions were recognized. Moreover, the pathways for the sequences assigned by SSR primers, the biological functions and gene interaction were determined. A total of 172,075 SSR motifs was identified on date palm genome sequence with a frequency of 450.97 SSRs per Mb. Out of these, 130,014 SSRs (75.6%) were located within the intergenic regions with a frequency of 499 SSRs per Mb. While, only 42,061 SSRs (24.4%) were located within the genic regions with a frequency of 347.5 SSRs per Mb. A total of 111,403 of SSR primer pairs were designed, that represents 291.9 SSR primers per Mb. Out of the 111,403, only 31,380 SSR primers were in the genic regions, while 80,023 primers were in the intergenic regions. A number of 250,507 SNPs were recognized in 84,172 SSR flanking regions, which represents 75.55% of the total SSR flanking regions. Out of 12,274 genes only 463 genes comprising 896 SSR primers were mapped onto 111 pathways using KEGG data base. The most abundant enzymes were identified in the pathway related to the biosynthesis of antibiotics. We tested 1031 SSR primers using both publicly available date palm genome sequences as templates in the *in silico* PCR reactions. Concerning *in vitro* validation, 31 SSR primers among those used in the *in silico* PCR were synthesized and tested for their ability to detect polymorphism among six Egyptian date palm cultivars. All tested primers have successfully amplified products, but only 18 primers detected polymorphic amplicons among the studied date palm cultivars.

## Introduction

Date palm (*Phoenix dactylifera* L., 2n = 36) [1] is one of the most economic important fruit trees in the Middle East and North Africa. It is a dioecious, cross pollinated, perennial monocotyledon and belongs of the order Arecaceae [2]. It has been cultivated in the Middle East from about 4000 B.C. [3]. There are over 2000 varieties that vary in shape, color, size, and weight [4]. Despite such a large number of varieties, for many years the detection of genetic variation in date palm was relying on the morphological variation between cultivars [5]. Morphological variations are uncertain and unstable because they could be affected by environmental factors, epistasis, pleiotropic effects and other factors. Therefore, as long as breeding programs depended on morphological markers alone, the pace of progress was bound to be slow. During the last few decades molecular markers were employed to detect the genetic polymorphism more accurately, and as useful tools in crop improvement and breeding programs. Among the most widely used molecular markers are amplified fragment length polymorphism (AFLP) [6], simple sequence repeats (SSRs) [7], randomly amplified polymorphic DNA (RAPD) and inter simple sequence repeats (ISSR) [8]. Compared with other types of molecular markers, SSRs have many advantages, such as simplicity, effectiveness, abundance, hyper variability, reproducibility, co-dominant inheritance and extensive genomic coverage [9].

In recent years date palm has been subjected to intensive genome sequencing studies. Yang et al. [10] reported the complete chloroplast genome sequence. The mitochondrial genome was assembled by Fang et al. [11] with an approximate length of about 715,001 bp. While, the first nuclear genome sequence of date palm was published by Al-Dous et al. [12]. It covered ~60% of the genome which represent ~380 Mb (25,059 genes) out of the 658Mb estimated genome size. They recognized more than 3.5 million polymorphic sites among the nine investigated varieties of date palm. Also, Al-Mssallem et al. [13] reported the second nuclear genome assembly which is about 605.4 Mb and covers 90% of the date palm genome.

Nowadays, the commonly published genome data and the availability of bioinformatics tools provide the scientists with tools to identify simple sequence repeats that could be used to develop useful SSR markers. Hamwieh et al. [7] surveyed the first Version (VS 01) of date palm genome and designed 1091 SSR primers. Zhao et al. [14] also successfully designed 4,967 EST-SSRs primers according to the available date palm expressed sequence tags (ESTs).

The present investigation was carried out aiming to: 1) identify and characterize, simple sequence repeats within the third Version (VS 3.0) of the date palm genome; 2) comparatively analyze genic and intergenic SSR motifs and develop a new SSR primers database; 3) recognize all single nucleotide polymorphisms (SNPs) that are located within the SSR flanking regions and 4) annotate the pathways for the sequences assigned by SSR primers and consequently determine the biological functions and gene interaction

## Materials and Methods

### Source of genomic sequences and SNPs Data

In this study, we used the Version 3.0 of the date palm draft genome sequence of Khalas female variety published by Al-Dous et al. [12]. It contains 57,277 scaffolds, about 381 Mb in length and covers about 60% of the genome sequence. Also, the 3,518,029 SNPs covering the whole genome were employed. All genome sequences and SNPs data were downloaded from http://qatar-weill.cornell.edu/research/datepalmGenome/download.html.

### SSR motifs mining and primer design

The Perl script MISA (MIcroSAtellite identification tool; http://pgrc.ipk-gatersleben.de/misa) and primer3_core [15] were used. These were inserted in the pipeline tool developed by the research team to identify and design SSR primers and to link it to SNPs that are located within the SSR flanking regions. A total of 57,277 scaffolds containing ~381 Mb sequences were used to determine SSR motifs with the following criteria: the repeat number in mononucleotide (mono-) ≥ 10 (bp), in dinucleotide (di-) ≥ 6 bp, in trinucleotide (tri-), tetranucleotide (tetra-), pentanucleotide (penta-), and hexanucleotide (hexa-) ≥ 5 bp. The maximal number of bases interrupting two SSR motifs in a compound microsatellite was 100 bp. SSR primers were designed to satisfy the following criteria: an optimum primer length of 20 bp, the optimum melting temperature of 55°C, the product size range of 100–500 bp, and a 50% optimum G/C content. The presence of SSRs was investigated in the genic and intergenic regions. These regions were determined according to previous classification of the genome sequences by Al-Dous et al. [12].

### Validation of the designed SSR primers

Two methods were used to validate our designed SSR primers. The first method was *in silico* validation by applying the bioinformatics tools to test 1031 SSR primer pairs using the primer search program:EMBOSS:6.5.7.0, among the two date palm genomes published by Al-Dous et al. [12] and Al-Mssallem et al. [13]. The first genome sequence was used to design our SSR primers which were given the name(PDK30 sequence) [12]. While, the second sequence is a whole genome shotgun sequence downloaded from the Genbank with BioProject: PRJNA83433 (http://www.ncbi.nlm.nih.gov) and designated as (ATBV01 sequence) [13].

*In vitro* validation was used as the second method. This was performed through designing and testing 31 SSR primer pairs, randomly selected from the 1031 primers previously tested *in silico*. *In vitro* test was applied to investigate the polymorphism among six Egyptian date palm cultivars (Zaghloul, Hayany, Samany, Barhee, Sewi and Bartamoda). Total genomic DNA was extracted from date palm leaves, using the DNeasy Plant Kit (QIAGEN). The PCR amplification reaction was prepared in 25 μl containing 50 ng of DNA, 2.5 mM MgCl_2_, 5 μl 5X PCR buffer, 0.5 mM each SSR primer, 0.5U of GoTaq Flexi DNA Polymerase, and 2.5 mM dNTPs. The PCR temperature profile was 95°C for 5 min, followed by 35 cycles of 95°C for 50 s, the annealing temperature for each SSR primer (shown on S1 & S2 Files) for 55 s, 72°C for 50 s, and the final extension step at 72°C for 10 min. The PCR products were checked and visualized on a 1.5% agarose gel in TBE buffer at 100 W for 120 min.

### Enzymes and pathways annotation

The Blast2GO program [16] was used to determine the enzyme codes (EC numbers) or enzyme annotations for 12,274 genes that contain 23,957 SSR primer pairs. The Blast2GO program provides EC annotation through the blast of all sequences against the NCBI database, then determine the gene ontology (GO) annotation. Consequently, the enzyme codes are highlighted on KEGG maps.

## Results and Discussion

The present study was based on the date palm genome sequences data published by Al-Dous et al. [12] and Al-Mssallem et al. [13].

### Distribution of the different SSR motifs in the date palm genome

Searching for SSRs in the PDK30 sequence [12] revealed that out of the examined 57,277 date palm scaffolds, 28,394 (49.5%) contain SSR repeats satisfying the chosen criteria for detecting SSRs. The number of identified SSR motifs was 172,075, with a frequency of 450.97 SSRs per Mb. The results of identified SSR motifs are shown in Table 1.

**Table 1:** Summary of the identified SSR motifs on the date palm PDK30 genome sequence.

Searching item	Results
**Total number of scaffolds examined**	57277
**Total size of examined sequences (bp)**	381563256
**Total number of identified SSRs**	172075
**Number of SSR containing sequences**	28394
**Number of sequences containing more than one SSR**	17237
**Number of SSRs present in compound formation**	22432

The SSRs motifs were not equally distributed in the genic and intergenic regions. Out of the 172,075 SSR motifs, 130,014 SSRs (75.6%) were located within the intergenic regions with a frequency of 499 SSRs per Mb. While, 42,061 SSRs (24.4%) were located within the genic regions with a frequency of 347.5 SSRs per Mb. Interestingly, Zhang et al. [17] surveyed the full-length of 30,854 genes generated from (assembled ESTs) of date palm, and identified 2,043 SSRs motifs. This number is lower than the number of SSRs identified in the present study. This could be due to the different criteria and the use of the full genome sequence adopted in our investigation.

Among the genic regions 1,785 SSRs were located in exons. This represents about 4.24% of the total genic regions (121018751 bp). However, 40,276 SSRs (95.76%) were located within the introns. The details of the identified SSRs in date palm are summarized in the S3 File.

**Fig 1:**
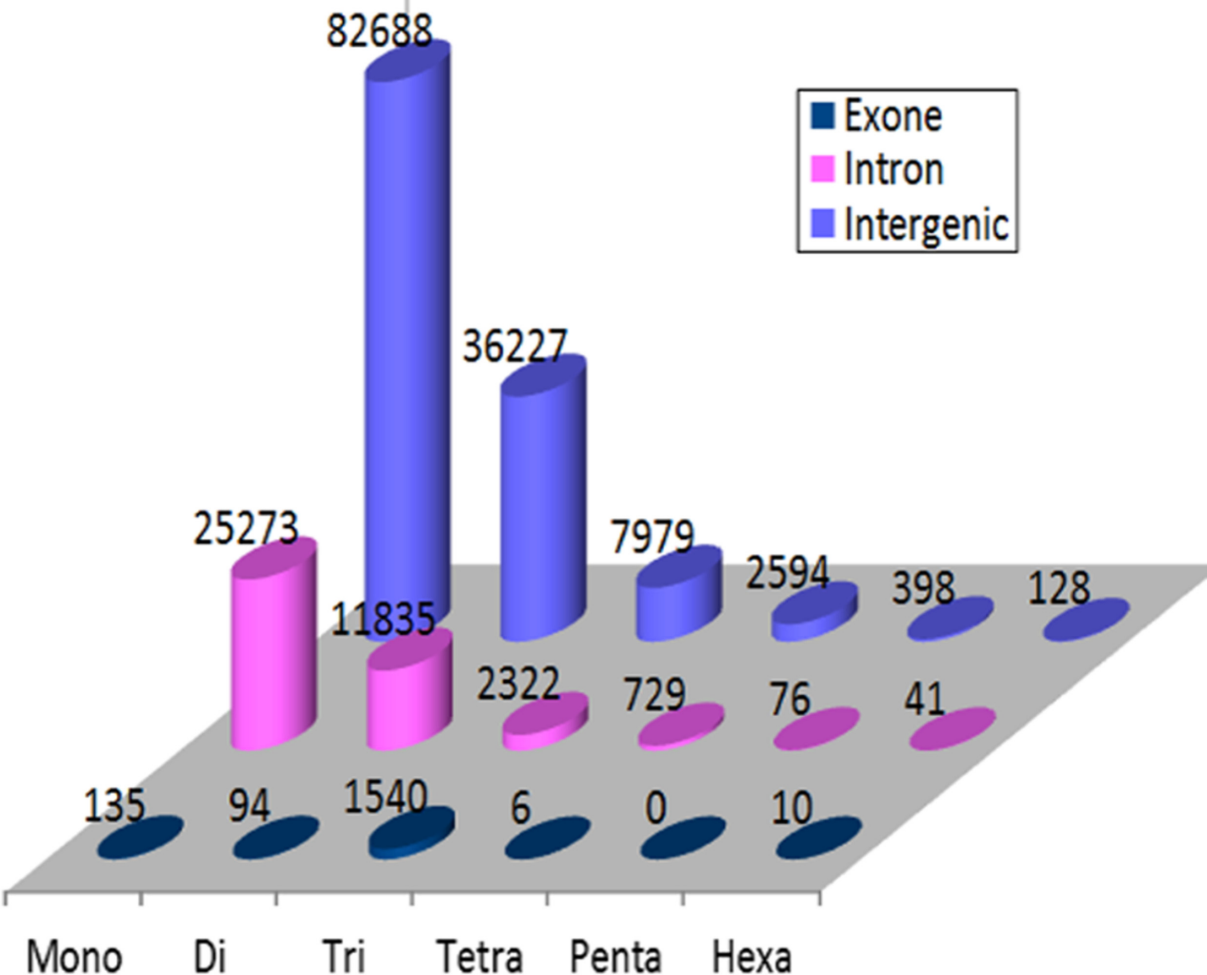
The Distribution of the various SSR classes in genic (introns and exons) and intergenic regions in date palm genome.

Our investigation of the different SSR motifs present in the intergenic region showed that the most abundant type was the mono- (63.6%), followed by the di- (27.8%), tri- (6.2%), tetra-(2%), penta- (0.3%) and hexa- (0.1%). However, in the introns of the genic regions the percentages of mono-, di-, tri-, tetra-, penta- and hexa- repeats were 62.7%, 29.3%, 5.8%, 1.9%, 0.2% and 0.1% respectively. While in the exons, the most frequent type was the tri-, followed by the mono-, di-, hexa- and tetra- repeats (86.2%, 7.6%, 5.3%, 0.6% and 0.3%, respectively). The penta- repeat was not found in the exon regions **(Fig 1)**.

Similarly, Zhao et al. [14] reported that the trinucleotide repeats were the most abundant in the exon regions. In general, the distribution of SSR motifs in the genic regions was consistent with Zhang et al. [17]. However, all the previous studies on date palm did not mention the presence of mononucleotide repeats [7,14,17]. To our knowledge this is the first investigation that shed light on the presence of the mononucleotide repeats in date palm.

The total population of the detected SSRs across the genome was classified as non-triplet repeats (mono, di, tetra and penta) and triplet repeats (tri and hexa). The results are summarized in Tables 2 and 3. As shown in Table 2, the frequency of the different non-triplet types with different repeat number was variable in the genic and intergenic regions. In general, there was a negative correlation between the number of repeats and the number of detected nontriplet motifs. Concerning the penta repeats, although the number was considerably lower in the genic than intergenic regions, however, they were confined to the 5–9 number of repeats.

**Table 2:** Comparison between non-triplet repeat types according to the number of repeats in both genic and intergenic regions.

Non-triplet								
	Mono-		Di-		Tetra-		Penta-	
No. of Repeat	Genic	Intergenic	Genic	Intergenic	Genic	Intergenic	Genic	Intergenic
**5–9**	-	-	8563	22859	726	2234	75	315
**10–15**	22944	68365	2408	5941	2	15	-	1
**16–20**	1810	6247	647	1584	1	3	-	-
**21–25**	433	1387	186	460	2	5	-	1
**26–30**	106	433	37	97	-	3	-	-
**31–35**	43	152	10	31	-	2	-	-
**36–45**	48	155	5	30	1	-	1	-
**46–55**	16	41	9	11	-	4	-	-
**56–70**	6	24	5	18	-	3	-	1
**71–100**	-	9	12	33	2	3	-	-
**101–150**	1	2	21	44	1	3	-	-
**151–200**	-	1	10	26	-	-	-	-
**201–350**	1	1	16	13	-	-	-	-
**Total**	25408	76817	11929	31147	735	2275	76	318

**Table 3:** Comparison between triplet repeat types according to the number of repeat in both genic and intergenic regions.

Triplet				
	Tri-		Hexa-	
No. of Repeat	Genic	Intergenic	Genic	Intergenic
**5–9**	3745	7259	49	115
**10–15**	75	152	-	1
**16–20**	12	21	-	-
**21–25**	4	10	-	1
**26–30**	-	4	-	-
**31–35**	-	2	-	-
**36–45**	1	-	-	-
**46–55**	2	3	-	-
**56–70**	3	5	1	-
**71–100**	4	8	1	1
**101–250**	16	6	-	-
**Total**	3862	7470	51	118

As shown in Table 3, the number of trinucleotide repeats decreased when the number of repeats increased from 5 to 35 repeats (from 3745 to 0 in the genic region and 7259 to 2 in the intergenic region). Afterwards these numbers started to increase but with very low frequency. While, the number of hexanucleotide repeats was considerably lower than all the other repeat types and they were confined to the 5 to 9 repeat number.

**Fig 2:**
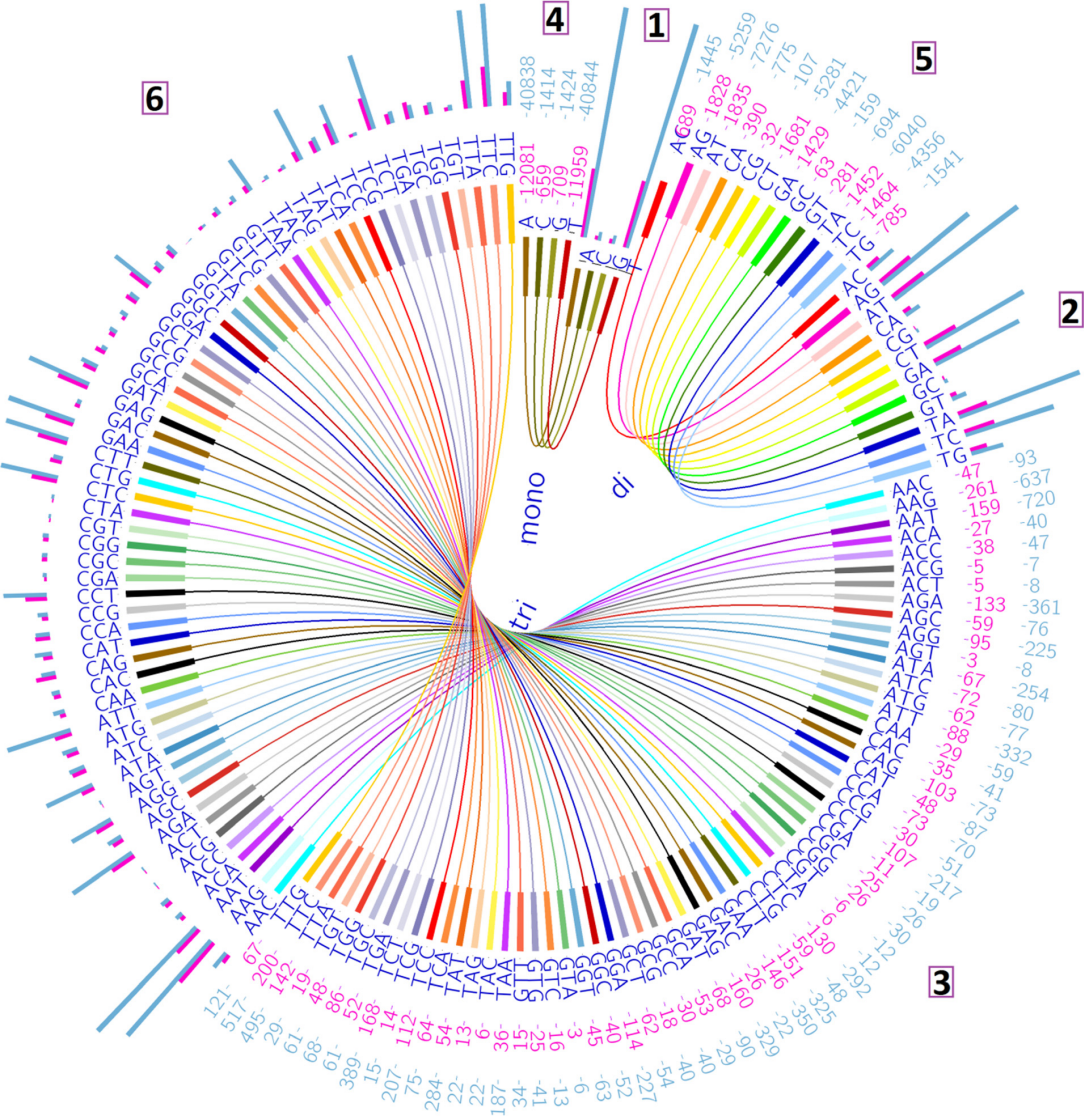
The comparison between mono, di and tri- repeats in both genic and intergenic regions. The numbers in boxes (1, 2, and 3) and (4, 5 and 6) represent the repeat types in the genic and intergenic regions, respectively. Similar repeats in the genic and intergenic regions are labeled with the same color. Numbers with pink color represent the number of repeats in the genic regions, while those with blue color refer to the intergenic regions. The latter data are illustrated with the same colors as histograms.

The high appearance of tri repeats in coding regions was consistent with previous studies in plant genomes [18–24]. **Fig 2** illustrates the mono, di and tri repeat sequences, number, and locations of the repeats whether in the genic or intergenic regions using the Circos software developed by Krzywinski et al. [25].

**Fig 3:**
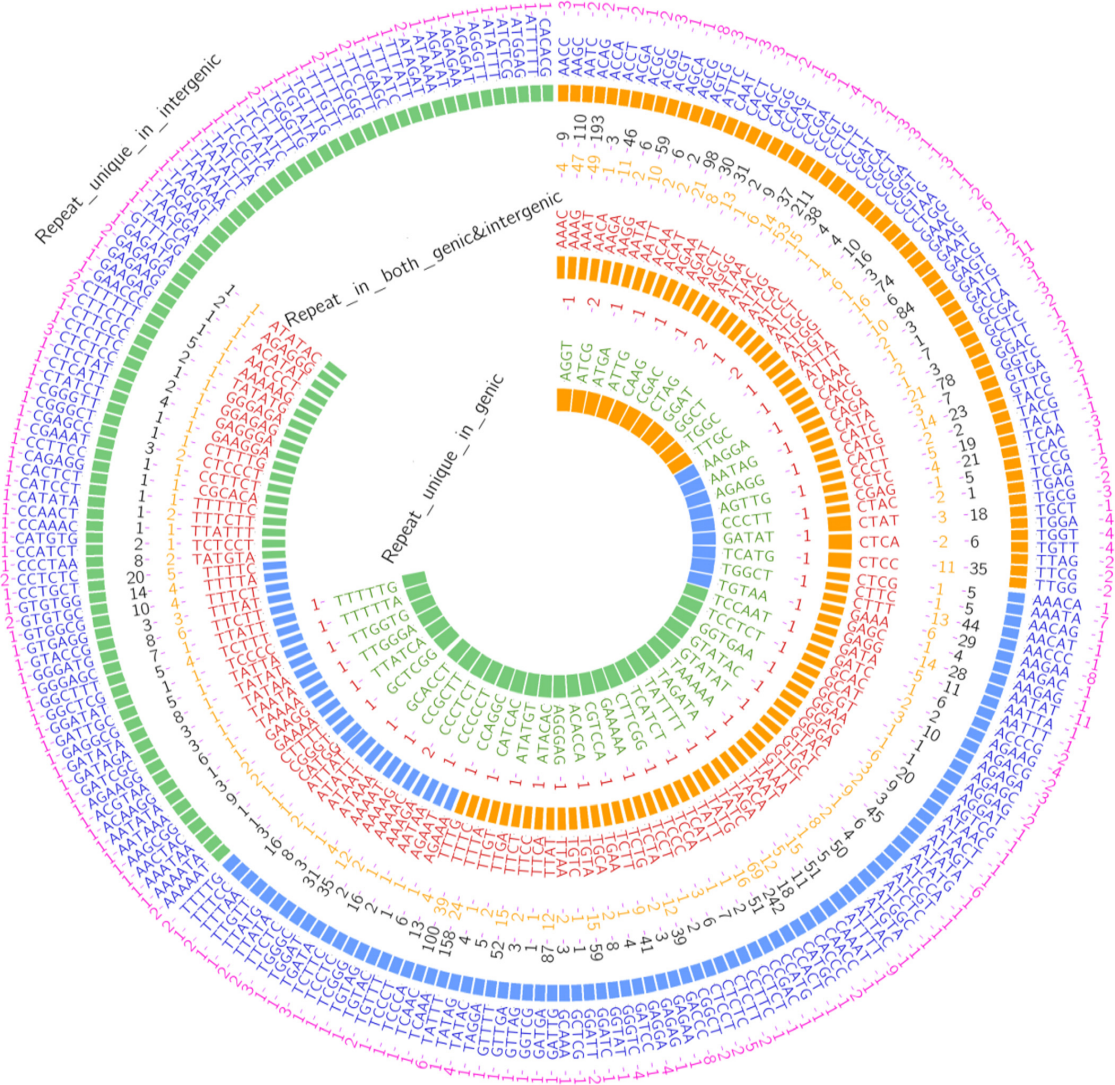
Comparison between tetra, penta and hexa- repeats in both genic and intergenic regions. The outer most circle in blue colored letters represents the different types of repeats in the intergenic regions. While, the pink colored numbers refer to the number of their respective repeats. The middle circle in red colored letters illustrates the types of repeats present in both genic and intergenic regions. While, the number of each repeat was yellow colored for the genic and black colored for the intergenic regions. The inner most circle with green colored letters shows the repeats unique to the genic regions, and the dark red colored numbers reveal the respective number of repeats. The orange, blue and green rectangles refer to tetra, penta and hexa repeats, respectively.

Concerning the other types of SSR repeats, 134 SSRs (tetra, penta and hexa) repeats were present in both genic and intergenic regions **(Fig 3).** While, 48 repeats were discovered in the genic regions only and comprised 11 tetra, 9 penta and 28 hexa repeats. In addition, 236 repeats were unique to the intergenic regions. The latter comprised 63 tetra, 84 penta and 88 hexa repeats. The present study represents the first report concerning the comparison of these specific SSR motifs (mono, di, tri, tetra, penta and hexa) in both genic and intergenic regions in the date palm genome.

Among the tri repeats, 60 different repeat sequences were determined Fig 2. The tri repeats are of special importance, since some of these repeats could represent coding sequences for amino acids, start or stop codons. This has also been suggested by Pinto et al. [26] and Kaur et al. [27]. They explained that trinucleotide repeats, can be accommodated more readily within coding regions, as changes in their length simply result in gain or loss of a single amino acid from a protein sequence.

In this context, Morgante et al. [20], Li et al. [28]and Kalia et al. [29] reported that in many species, triplet SSRs are the most abundant repeats in the exons. They claimed that this could be due to the suppression of non–trimeric SSRs in coding regions which could be the result of change in reading frame with increase or decrease in number of repeat units and the negative selection against frame shift mutations in coding regions. In the present study, the most frequent tri-repeat was AAG as its frequency was 261 in the coding regions. This finding is in good accordance with Li et al. [28].They mentioned that in plants, the most frequent triplet is AAG. However, [30–32]found that in cereals, the most common triplet is CCG. Moreover, [29,33]mentioned that dicots have more mononucleotide repeats and monocots have more trinucleotide repeats.

In non-coding regions the rate of base substitution is high [29], however it could also occur in coding regions. Therefore, we surveyed all tri repeat types in the exons to detect probable substitutions that could convert an amino acid to a stop codon. Seventeen repeat sequences were detected Table 4.

**Table 4:** Different base substitution probabilities that may convert tri repeats in date palm exons to stop codons.

Amino acid	Repeat Sequence	Stop Codon	Amino acid	Repeat Sequence	Stop Codon
**Arginine**	CGA	TGA	**Cysteine**	TGT	TGA
	AGA	TGA		TGC	TGA
**Glycine**	GGA	TGA	**Tryptophan**	TGG	TGA
	GAG	TAG	**Glutamine**	CAG	TAG
**Leucine**	TTA	TGA		CAA	TAA
	TTG	TAG	**Lysine**	AAA	TAA
**Serine**	TCA	TGA		AAG	TAG
	TCG	TAG	**Tyrosine**	TAC	TAA
**Glutamic acid**	GAA	TAA		TAT	TAA

### SSR primers Design

**Fig 4:**
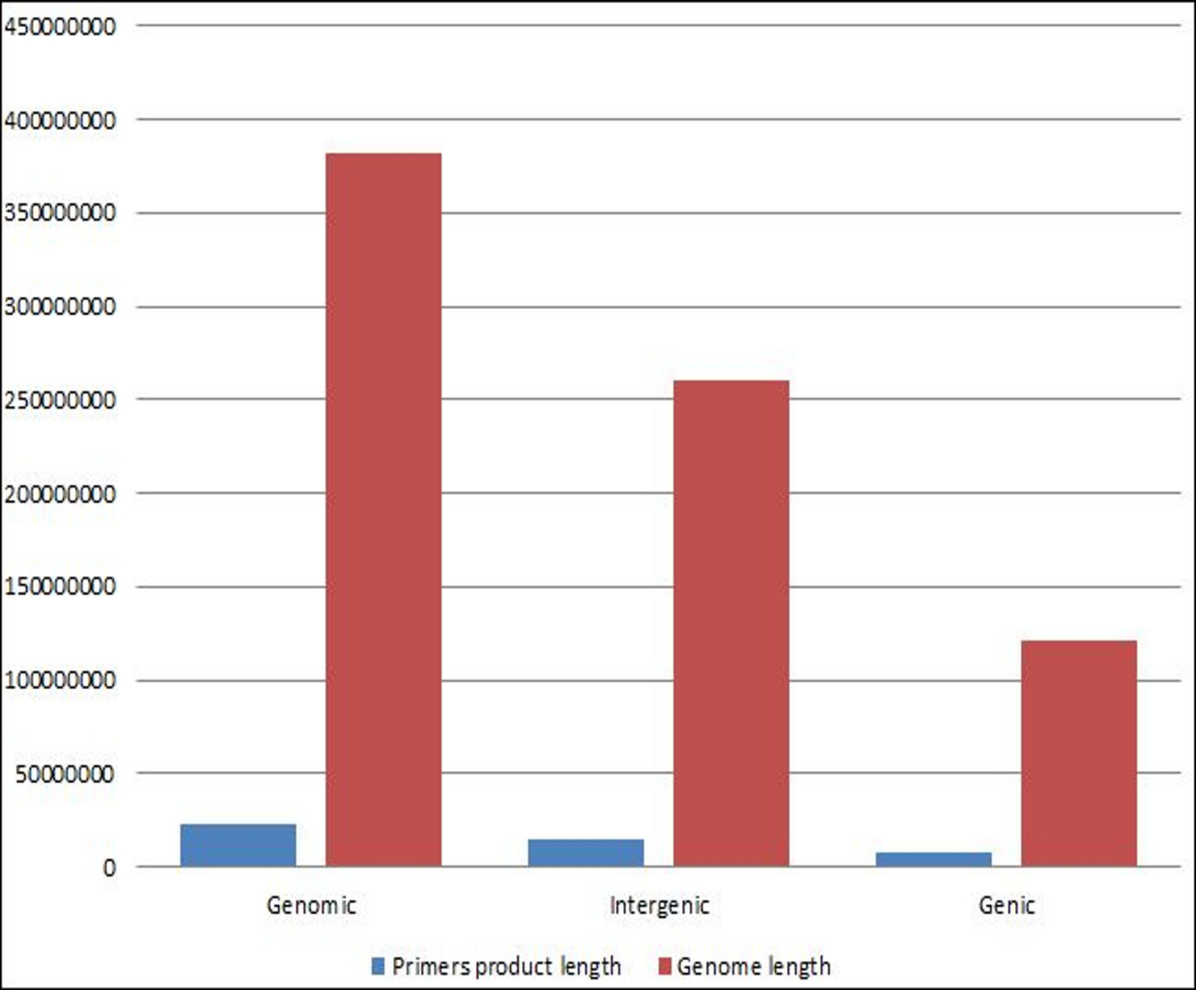
Comparison between primers product length within genic and intergenic region on date palm genome. The first column (left) represents the total genome length and total length of primers product, and the other two columns represent total length of primers product comparing to the total length of genic and intergenic regions.

A total of 111,403 SSR primer pairs were designed, that represents 291.9 SSR primers per Mb and covers the amplification of 22,652,015 bp (5.9%) of the total genome. Out of the 111,403, only 31,380 SSR primers were developed in the genic regions. These primers could amplify about 7,727,736 bp (6.38%) of the total genic regions. However, 80,023 primers covered the amplification of about 14924279 bp (5.72%) of the total intergenic regions **(Fig 4).**

Using the Version 1.0 date palm genome, Hamwieh et al. [7] designed only 1091 SSR primers. However, in the present study, we used Version 3.0 of date palm genome and succeeded to design 111,403 SSR primers in both genic and intergenic regions. To distinguish between our SSRs and other SSR primers developed on date palm genome, such as those previous developed [14,34,35], we named the Genic and intergenic SSR primers as Pd_G_SSRx and Pd_IG_SSRx, respectively (where Pd_G_SSR refers to *Phoenix dactylifera* Genic SSR primers, and Pd_IG_SSR was refers to *Phoenix dactylifera* Intergenic SSR primers and x refers to serial number).

### Determination and Distribution of SNPs located within SSRs flanking regions

**Fig 5:**
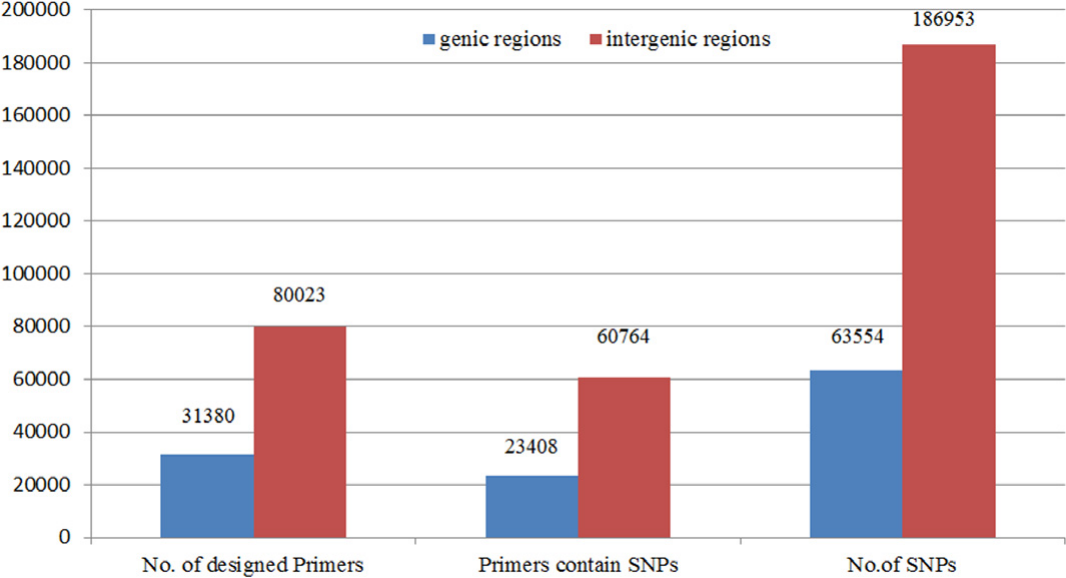
Comparison between the number of SSR primers and the number of detected SNPs within the genic and the Intergenic regions in the date palm genome.

SNPs data were downloaded from http://qatarweill.cornell.edu/research/datepalmGenome/download.html, and were subjected to Perl script developed by our research team to recognize all SNPs located in the SSR flanking regions. A total of 250,507 SNPs were determined in 84,172 SSR regions which represent 75.55% of the total SSR flanking regions. Therefore, the frequency of SNPs per SSR flanking region was 2.97. The distribution of these SNPs among the genome revealed that a number of 186,953 SNPs was located in 60,764 intergenic SSR flanking regions, while 63,554 SNPs were located in 23,408 genic SSR flanking regions **(Fig 5).** Thus, the high range of variation among the date palm genotypes could be attributed to the high record of SNPs in the genic regions.

### Development of date palm SSR primers database

The newly designed SSR primer pairs and related SNPs within the genic and intergenic regions were listed in the (S1 and S2 Files, respectively representing the two different databases). Each database contains information concerning the name of primers, repeats type, repeats sequence, repeats size, repeats start and end in the amplified sequences, forward and reverse primer sequences, primers annealing temperature, GC%, product size, corresponding genomic sequences, contig ID within date palm genome, gene product, gene ID (start and end on genome) and position of SNPs within SSRs flanking region. In addition, the gene product and SNPs location as designated by Al-Dous et al. [12] have been also incorporated in the developed database.

### Detection of SSRs in disease resistance related genes

The developed genic database was exploited to recognize the designed SSR primer pairs within the disease resistance related genes. A total of 42 SSR primer pairs were located within 16 different genes related to disease resistance. These results are summarized in Table 5.

**Table 5:** SSR primers designed within disease resistance related genes.

Gene product ^1^	contig ID ^2^	Gene Start ^3^	Gene End ^3^	Primer name ^4^
**Brown planthopper-induced resistance protein 1**	PDK_30s1053751	12041	18193	Pd_GSSR1733
	PDK_30s1160101	6656	7974	Pd_GSSR4967
**CC-NBS-LRR resistance protein**	PDK_30s779571	1328	9575	Pd_GSSR14611
	PDK_30s1078571	2782	7044	Pd_GSSR24273
	PDK_30s790641	783	4086	Pd_GSSR28574
	PDK_30s1108901	7116	10724	Pd_GSSR3477
	PDK_30s1130951	4900	9698	Pd_GSSR4096
	PDK_30s1159381	18412	23365	Pd_GSSR4958, Pd_GSSR4959
**Disease resistance protein rps2**	PDK_30s676021	11551	15125	Pd_GSSR8887
**Enhanced disease resistance 1**	PDK_30s1065091	52197	62496	Pd_GSSR2000, Pd_GSSR2002
**Mlo family protein**	PDK_30s998451	515	10639	Pd_GSSR31369
**Mlo-like protein 14**	PDK_30s665521	33248	35690	Pd_GSSR26227, Pd_GSSR26229
**Natural resistance-associated macrophage protein**	PDK_30s853031	786	7042	Pd_GSSR17867, Pd_GSSR17868
**NBS-LRR type resistance protein**	PDK_30s742701	1713	6858	Pd_GSSR12867
	PDK_30s665281	87243	102914	Pd_GSSR8157, Pd_GSSR8161, Pd_GSSR26210,Pd_GSSR26212, Pd_GSSR26213
**Non-race specific disease resistance 1**	PDK_30s909521	479	7523	Pd_GSSR20333, Pd_GSSR20334
**Protein kinase xa21**	PDK_30s866171	2129	11697	Pd_GSSR18479, Pd_GSSR18481, Pd_GSSR29704
	PDK_30s981471	9730	21118	Pd_GSSR22786,Pd_GSSR22787, Pd_GSSR22790, Pd_GSSR22791
**Rin13 (rpm1 interacting protein 13)**	PDK_30s1036661	1243	5906	Pd_GSSR1055, Pd_GSSR1058, Pd_GSSR1061
**Rpm1-interacting protein 4**	PDK_30s866791	3182	7027	Pd_GSSR29709
**Rust resistance kinase lr10**	PDK_30s767921	186922	194251	Pd_GSSR14032, Pd_GSSR14033, Pd_GSSR14034
**Seven transmembrane protein mlo8 (mlo8)**	PDK_30s827291	2600	6751	Pd_GSSR16802
**TIR-NBS resistance protein**	PDK_30s1076091	57384	60783	Pd_GSSR2418
**TIR-NBS-LRR type disease resistance protein**	PDK_30s716241	11276	14302	Pd_GSSR27256

^**1**^ **disease resistance genes product,**

^**2**^ **contig ID in date palm genome,**

^**3**^ **gene start and end in date palm genome,**

^**4**^ **primer name in our data base.**

### Validation of the designed primers

To validate our designed SSR primers, the first test was *in silico* PCR. We tested 1031 SSR primers using the primersearch program: EMBOSS: 6.5.7.0. Both publicly available date palm genome PDK Version 3.0 (PDK30) sequence [12] and ATBV01 sequence [13]were used as templates in the *in silico* PCR reactions. When using the date palm genome PDK30 sequence[12], all the 1031 SSR primer pairs successfully found 2450 complementary sequences with an average number of 2.37 alleles. However, only 903 SSR primers could successfully hit with 2774 complementary sequences in ATBV01 sequence [13] with an average number of 2.69 alleles. These results are summarized in (Table 6 and S4 File).

**Table 6:** Summary statistics of the *in silico* validated detected SSR in date palm genome sequences.

Items	PDK30 sequence^1^	ATBV01 sequence^2^
**No. of tested primers**	1031	1031
**No. of primers found hits in genome sequence**	1031	903
**No. of hit sequences in genome**	2450	2774
**Total length of hit sequences in genome(bp)**	613364	696699

^1^PDK30 sequence (Al-Dous et al [12])

^2^ATBV01 sequence (Al-Mssallem et al [13])

**Fig 6:**
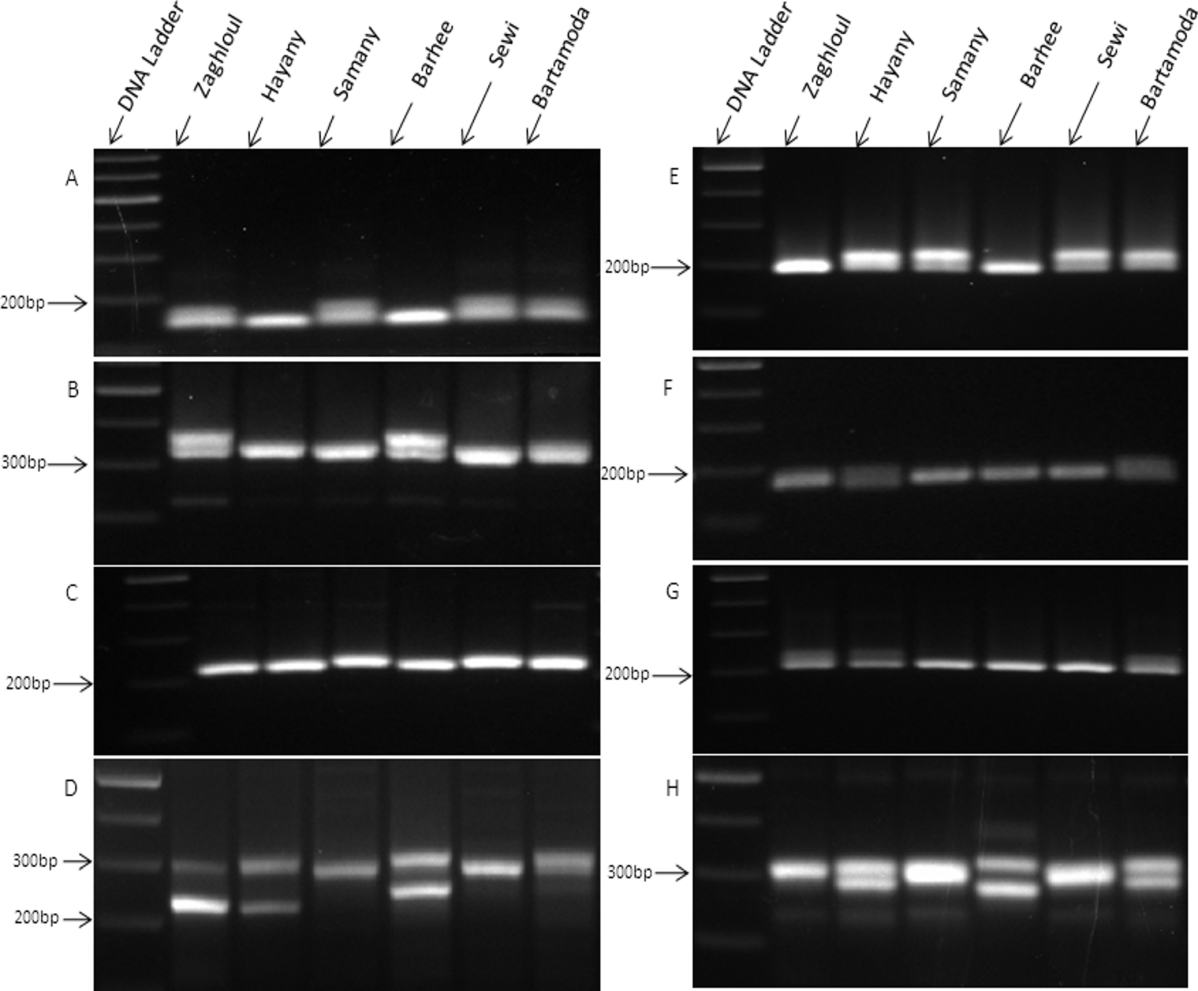
*In vitro* PCR analysis of the genomic DNA from six date palm cultivars using eight designed SSR primer pairs (A-H).

Concerning the *in vitro* validation, 31 SSR primers among those used in the *in silico* PCR were synthesized and tested for their ability to detect polymorphism among six Egyptian date palm cultivars. All tested primers have successfully amplified products, but only 18 primers detected polymorphic amplicons among the studied date palm cultivars **(Fig 6).**

The comparisons between the results of the *in silico* and *in vitro* PCR are summarized in Table 7. The results from both *in silico* and *in vitro* PCR were consistent in the number and length of alleles for some primers, while other primers recorded polymorphism in fragments sequence and differences in some alleles length. Moreover, some alleles could be detected in one genome and absent in other genome sequence. As shown in Table 7, out of the 104 alleles detected *in vitro* only 23 were consistent in both *in silico* and *in vitro* PCR.

**Table 7:** The comparison between the summarized results of the *in silico* and *in vitro* PCR.

*In vitro* PCR									*In silico* PCR		
			Zaghloul	Hayany	Samany	Barhee	Sewi	Bartamoda		Khalas (PDK30) ª	Khalas (ATPV01) ᵇ
Primer name	Forward & Reverse primer sequence	No. of alleles	Allele length (bp)	Allele length (bp)	Allele length (bp)	Allele length (bp)	Allele length (bp)	Allele length (bp)	No. of allele	Allele length (bp)	Allele length (bp)
**Pd_GSSR14034**	CGCACTCTGGAATCAACTCACATCTGCCGACAGAGTCAAA	1	143 ª	143ª	143ª	143ª	143ª	143ª	2	143ª	142
**Pd_GSSR18389**	AGGCAGAGAGATGTCCGTGTAAAAGTCTCCATGTCCGTGC	1	301ªᵇ	301ªᵇ	301ªᵇ	301ªᵇ	301ªᵇ	301ªᵇ	1	301ª	301ᵇ
**Pd_GSSR18525**	CATGCCTGCTATGATCGGTACAGGAAGATCCCAAATCGAA	2	200	200/222	200/222	200	200/222	200/222	2	191	244
**Pd_GSSR18997**	GCCTGATGATAGTCTCGGCTGCGAACCATACGAAAGCAGT	2	173ªᵇ	150	173ªᵇ	ND	173ªᵇ	150	2	160/173ª	160/173ᵇ
**Pd_GSSR19717**	CCATTTCGATGTCACCTCCTGCGAACCATACGAAAGCAGT	1	229ªᵇ	229ªᵇ	229ªᵇ	229ªᵇ	229ªᵇ	229ªᵇ	1	229ª	229ᵇ
**Pd_GSSR19798**	AGTCGTGGAGAGATTGCGTTGCCCATCTTGTAGGGAGACA	3	328/354	328	328	328/354	315	328	2	333	274
**Pd_GSSR19852**	CGGAAGATGCTGAAGTCTCCGCCATAGGAGTTTCAGTCGG	2	220ªᵇ/434	220ªᵇ	220ªᵇ/434	220ªᵇ	220ªᵇ	220ªᵇ/434	1	220 ª	220ᵇ
**Pd_GSSR2002**	TTTGGGATGTTGAATGGGTTTCTTTGGTGGAAAAAGCCAG	1	340ªᵇ	340ªᵇ	340ªᵇ	340ªᵇ	340ªᵇ	340ªᵇ	2	340ª/413	340ᵇ/413
**Pd_GSSR20333**	AAGTTCATGGGATGGTCGAGGGCTTCAACAATATGCGACA	8	231/562/674	239/674	247/562/674	239/562	247/562/674	243/674	2	225/227	227
**Pd_GSSR21118**	AGGTGGAGGCCTTCATAGGTTGAATTTGTGCTAGCGATGC	1	257ªᵇ	257ªᵇ	257ªᵇ	257ªᵇ	257ªᵇ	257ªᵇ	1	257ª	257ᵇ
**Pd_GSSR22331**	TACGTGGTCTTGCACGGTAATTAAGCTCGCACTCCTCGAT	2	213ªᵇ/238	213ªᵇ/238	213ªᵇ	213ªᵇ	213ªᵇ	213ªᵇ/238	1	213ª	213ᵇ
**Pd_GSSR2418**	ACTCCCATGTAAACCTCCCCATGTGGGTTGGGTTTGTTGT	1	237ª	237ª	237ª	237ª	237ª	237ª	3	237ª	236/613
**Pd_GSSR3388**	TTCCAATGAAAGCCTTTTGGACCCGGAACAGGTTACTGAG	4	165	165	171	171	178	178	2	155	152
**Pd_GSSR3981**	TTCCTCCTGTTTTTCCCCTTCTCACCGGCTCTACCAGAAG	1	278ª	278ª	278ª	278ª	278ª	278ª	2	278 ª	277
**Pd_GSSR4096**	CGCAATCATTAAGCTCAGTCAGTTGGGAATGGGTAAGGTCAA	1	315	315	315	315	315	315	2	329	328
**Pd_GSSR4657**	TTGATGAGCCTCCTCTTTGGGATGGTGAGAGTTGGGGAGA	3	333ªᵇ/740	333ªᵇ/740	333ªᵇ/740	333ªᵇ/337/740	333ªᵇ/740	333ªᵇ/740	1	333ª	333ᵇ
**Pd_GSSR4967**	TGGCCATCGAGTGCTACATAAGGCTTCGTTCCTCCAACTT	2	200/586	200/586	200/586	200/586	200/586	200/586	1	189	189
**Pd_GSSR6184**	CCCCAGAAAATGCCTTAACAAAGAGCGTTGACTGCTACCAA	1	125ªᵇ	125ªᵇ	125ªᵇ	125ªᵇ	125ªᵇ	125ªᵇ	1	125ª	125ᵇ
**Pd_GSSR7241**	GATGCCAAGCACTGTGATGTTATCCTGCATGCACCAATGT	1	348ªᵇ	348ªᵇ	348ªᵇ	348ªᵇ	348ªᵇ	348ªᵇ	3	229/348ª	229/348ᵇ/397
**Pd_GSSR8157**	CAGCTCTCGGGAAATCTTTGTGCCACTGTTTTTGGATCAG	2	165ªᵇ	165ªᵇ/186ªᵇ	165ªᵇ	165ªᵇ	165ªᵇ	165ªᵇ/186ªᵇ	2	165ª/186ª	165ᵇ/186ᵇ
**Pd_IG_SSR_123**	TTGCTAGAACCCTAACCCCCCCCAACCCGTTTAAGGAAAT	7	497/539	488/533	505	484	484	474	5	284/388/420/714/769	ND
**Pd_IG_SSR_18**	GAAACGGGCCCCTAGAATTATCACTGTCTCCACCACCATC	14	198/282/329	198/282	188/352	179/264/307	171/220/252/331	163/248	3	287	214/291
**Pd_IG_SSR_211**	TGGATGTTTCTGGTTACTGTTGTTAGGAACCCCCTTATCCCA	4	256ª	278	256ª	278/253ᵇ	256ª	267	2	356ª	253ᵇ
**Pd_IG_SSR_289**	GGCACTCCATGACCTTTTGTAAACAAGCCGAAACCAACAG	2	225ᵇ	225ᵇ	240ª/225ᵇ	225ᵇ	225ᵇ	229/240ª	3	152/240 ª	152/225ᵇ
**Pd_IG_SSR_343**	TGCAACAAAGAGATCTGCCAAGACAAAGGCTTCCCCAAAT	6	304ª	282/304ª	304ª	274/316	295	282/312	3	195/231/304ª	195/231
**Pd_IG_SSR_38**	CGCGGTCACTGAAGTCAATAGGAAACCCATGGGAACATAA	6	224/295	224/300	290	250/309	290	309	1	326	ND
**Pd_IG_SSR_500**	GCGGCATCCTCTTGAACTTATTTCCAATCCAACCTAGCAGTT	7	266	266/319	261	254/280/314	254	254/319/343	2	271/333	271
**Pd_IG_SSR_539**	TGGTTCAGGAGAAGCATGTGGAAGAAATTGGGAGAATTAGGG	2	278/343	278/343	278/343	278/343	278/343	278/343	2	258/357	258
**Pd_IG_SSR_619**	CTGCATGACTTGGCACCTTAAAGGCCTTAGCCCAAAGAAG	6	210/240ª/270	210/265	210/236/260	210/240ª/265	210/240ª/270	210/265	1	240ª	ND
**Pd_IG_SSR_65**	CCTCTTATCCTTCTCTTCGGGAACTTTCTTCTGCATTGCCA	2	354/428	354/428	354/428	354/428	354/428	354/428	1	488	ND
**Pd_IG_SSR_660**	TAGATCCTCCCCTTTACCCGGTATACACACACACGCACGC	8	150ªᵇ/168	150ªᵇ	157/180	159	168/198ᵇ	168/190	2	150ª	150ᵇ/298ᵇ

^a^ The bands consistent with Khalas (PDK30 sequence) [12]

^b^ The bands consistent with Khalas (ATPV01 sequence) [13], ND non detected.

### Functional classification by KEGG pathway analyses

The Kyoto Encyclopedia of Genes and Genomes (KEGG) pathway database is a useful tool for understanding genes biological functions and their molecular interactions[36]. We used the Blast2GO program [16] to determine the Enzyme (EC numbers) or Enzyme annotations for 12,274 genes that contain 23,957 SSR primers. The Blast2GO program provides EC annotation through the blast of all sequences against the NCBI database, then determine the gene ontology (GO) annotation, consequently, the enzyme codes are highlighted on KEGG maps.

Based on the basic parameters of the Blast2GO program, the blast results revealed that 3787 sequences were partial genes, thus, this number was excluded from the next steps of analysis. The remaining 8487 sequences showed blast result path to gene ontology (GO) annotation step. This step indicated 3201 sequences with no GO terms, while, 5286 sequences were assigned to 13554 GO terms, ranging from 1 to 45 GO terms per sequence. Sequences with GO terms were introduced into the EC number detect step. This analysis revealed only 463 unique sequences (genes), which encode an actual number of 258 enzymes distributed among 111 different pathways. These genes comprised 896 SSR primers because each gene sequence might contain more than one SSR primer. The sequences with blast results, GO terms and EC numbers were listed in S5 File and the position of these genes within the genome and the SSR primers name for each gene were listed in S6 File.

**Fig 7:**
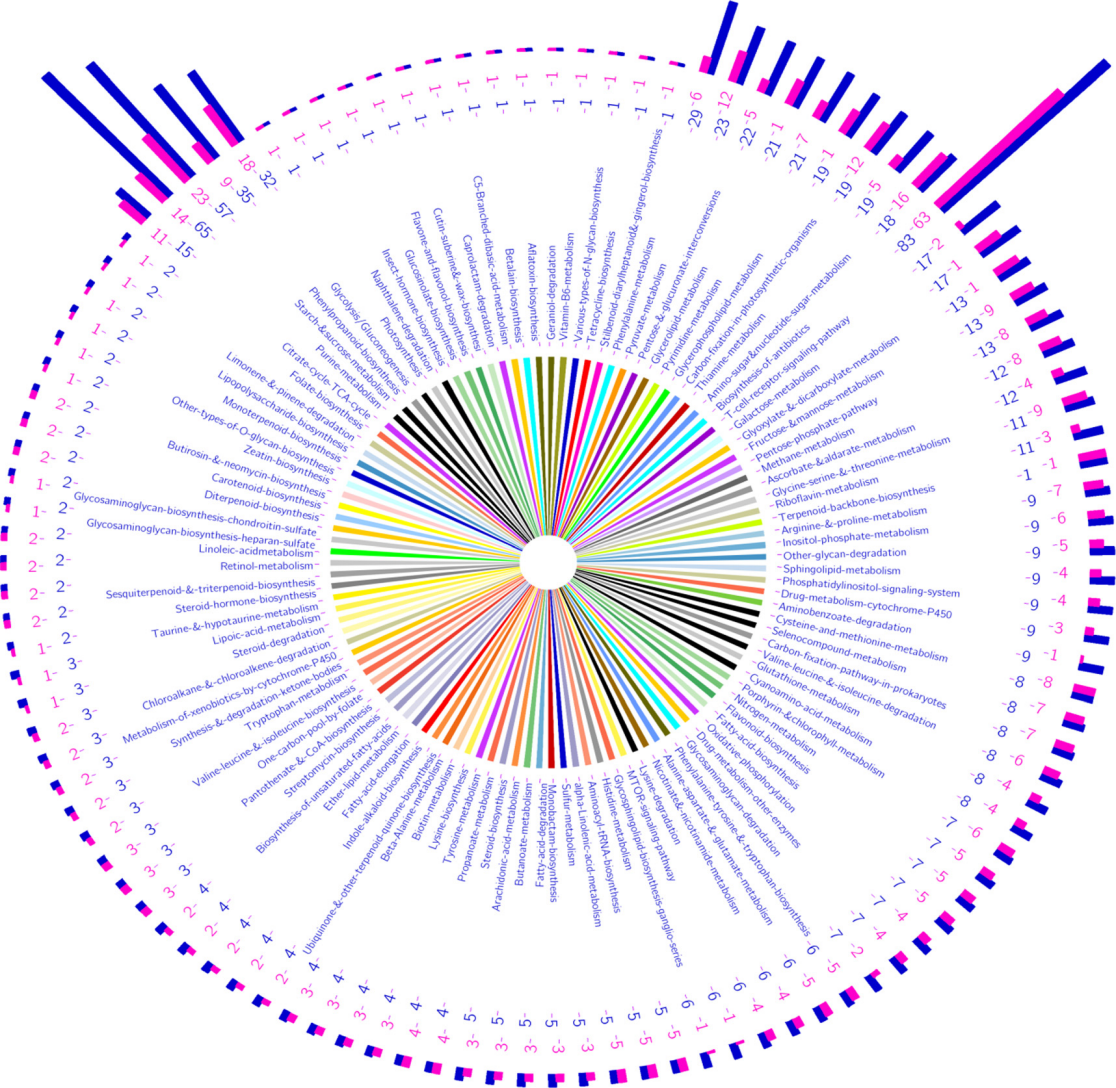
Pathways assigned using the designed SSR primers. The middle part of the figure shows the pathways name. The outer part of the figure reveals the histogram, number of enzymes and number of genes encoding the enzymes in each pathway (where the purple color refers to the number of enzymes in the pathway and the blue color refers to the number of genes encoding the enzyme).

The data for the pathways name, name of each enzyme and the position of their coding genes within the genome, in addition to the EC numbers for each enzyme were listed in S7 File.The most abundant enzymes were identified in the pathways related to: (1) the biosynthesis of antibiotics (63 enzymes encoded by 83 genes); (2) starch and sucrose metabolism (23 enzymes encoded by 57 genes); (3) glycolysis/Gluconeogenesis (18 enzymes encoded by 32 genes) and (4) amino sugar and nucleotide sugar metabolism (16 enzymes encoded by 18 genes). While, only one enzyme encoded by one gene was identified in the pathway related to insect hormone biosynthesis. The detected pathways, the number of enzymes and number of genes encoding the enzymes represented as histograms, are illustrated in **(Fig 7).**

**Fig 8:**
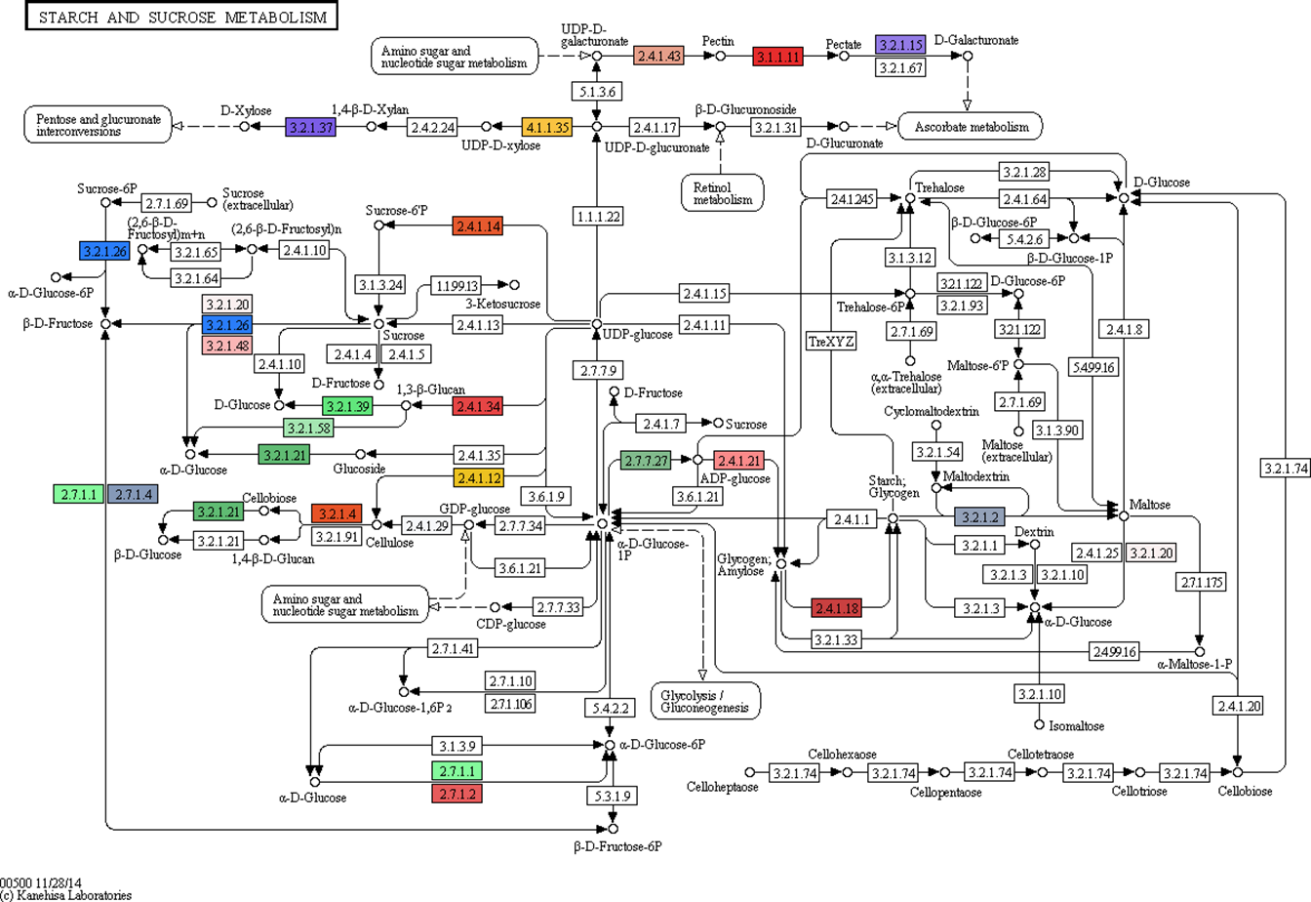
The annotation of the 23 enzymes assigned using our SSR primers in the starch and sucrose metabolism pathway as revealed by the Blast2GO program. Each enzyme code is highlighted with different color.

**Fig 9:**
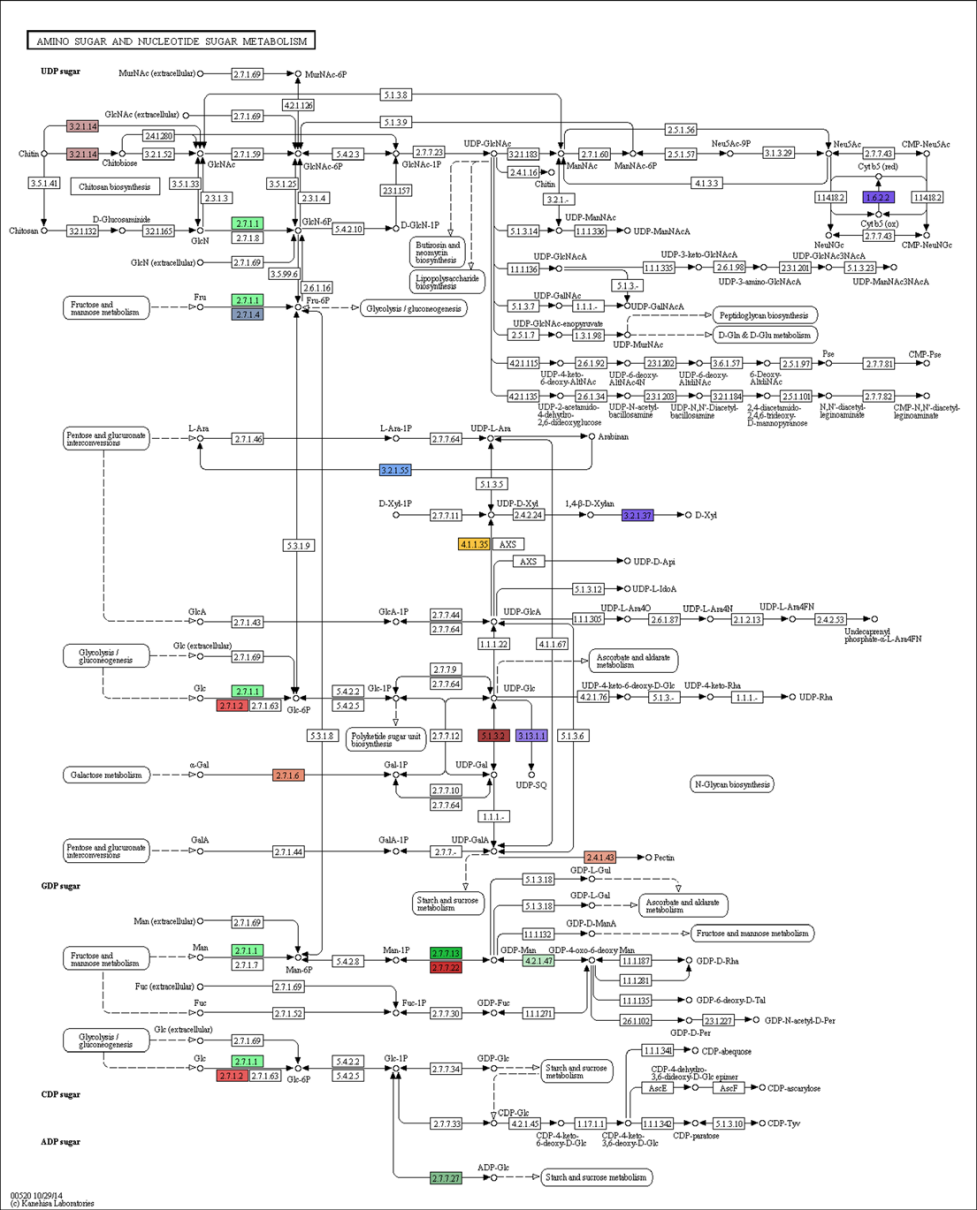
The annotation of the 16 enzymes assigned using our SSR primers in the amino sugar and nucleotide sugar metabolism pathway as revealed by the Blast2GO program. Each enzyme code is highlighted with different color.

To confirm the pathway annotation results, two pathways, i.e., the starch and sucrose metabolism and the amino sugar and nucleotide sugar metabolism pathways were selected to apply the Blast2GO program. This program highlighted the identified Enzyme codes on the respective KEGG map as shown in **Figs 8 and 9,** respectively.

## Conclusion

This study extends the basic research on date palm in an attempt to promote applying genomics and bioinformatics to enhance breeding and improvement programs of this important crop. The comparative studies depending on SSR motifs are useful since they represent co-dominant markers, highly polymorphic regions and could be used in genetic diversity analysis and breeding programs through designing specific primers for these regions. Genic SSR primers are helpful to detect the polymorphism within functional regions. Therefore, the breeder can choose the appropriate primers according to their localization in the biological pathways related to the trait of interest. Moreover, SNPs in the SSR flanking regions could provide an additional advantage to the breeders. Furthermore, this investigation highlights the usefulness of the *in silico* PCR for testing the primers before their use *in vitro*, thus saving time, effort and cost.

## Supporting Information

S1 File The newly designed SSR primer pairs and related SNPs within the genic regions. (XLSX)

S2 File The newly designed SSR primer pairs and related SNPs within the intergenic regions. (XLSX)

S3 File The details of the identified SSRs in date palm genome. (XLSX)

S4 File The summarized results from the *in silico* PCR reactions. (XLSX)

S5 File List of the sequences with blast results, GO terms and EC numbers. (XLSX)

S6 File The position of the genes that encoded to enzymes within the genome and the SSR primers name for each gene. (XLSX)

S7 File The list of the data for the pathways name, name of each enzyme and the position of their coding genes within the genome, in addition to the EC numbers for each enzyme. (XLSX)
